# Supplementary material for: Defect-Enriched Graphene Nanoribbons Tune the Adsorption Behavior of the Mediator to Boost the Lactate/Oxygen Biofuel Cell
Source: Nanomaterials (Basel). 2023 Mar 17;13(6):1089. doi: 10.3390/nano13061089 (PMC10058110; doi:10.3390/nano13061089)
Supplement: Supplementary file 1 [file nanomaterials-13-01089-s001.zip › nanomaterials-2260175-supplementary.pdf]

Supporting Information

Defect-Enriched Graphene Nanoribbons Tune the Adsorption Behavior of the Mediator to Boost the Lactate/Oxygen Biofuel Cell

Xiaoyu Feng [1,2,†], Yongyue Ning [2,3,†], Zhongdong Wu [2], Zihan Li [2,3], Cuixing Xu [2], Gangyong Li [2,4,*], and Zongqian Hu [2,*]

1 College of Textiles and Clothing, Xinjiang University, Urumqi 830046, China; xiaoyuf1997@163.com
2 Beijing Institute of Radiation Medicine, Beijing 100850, China; 15543658978@163.com (Y.N.); 5226430016@stu.ecnu.edu.cn (Z.W.); lzh1457455119@163.com (Z.L.); xucx061@nenu.edu.cn (C.X.)
3 Key Laboratory of Nanobiosensing and Nanobioanalysis, Universities of Jilin Province, Northeast Normal University, Changchun 130024, China
4 Key Laboratory of Hunan Province for Advanced Carbon-Based Functional Materials, School of Chemistry and Chemical Engineering, Hunan Institute of Science and Technology, Yueyang 414006, China
* Correspondence: huzongqian@hotmail.com (Z.H.); ligangyong117@163.com (G.L.)
† These authors contributed equally to this work.

Table S1. List of properties of tear-based EBFCs.

Bioanode	Biocathode	Biofuel	OCV (V)	J (μA/cm^2)	P (μW/cm^2)	Ref.
AuNPs/ CDH	AuNPs/ MvBOx	Human tears (0.05mM glucose)	0.57	n/a	1	[1]
AuNPs/TTF-TCNQ	AuNPs/ BOx	Human tears (0.665mM ascorbate)	0.54	n/a	3.1	[2]
BP/poly-MG/ LDH/NAD$^+$	An-pyr-MWCNT/TBAB-modified Nafion/BOx	Artificial tear (3mM lactate)	0.413±0.06	61.3±2.9	8.01±1.4	[3]
LOx/ FcMe$_2$-LPEI	An-Pyr-MWCNT/TBAB-modified Nafion/BOx	Artificial tear (3mM lactate)	0.44±0.08	22±4	2.4±0.9	[4]
NPG/Os(bpy)$_2$ PVI/LOx	NPG-diazonium-BOx	Artificial tear (3mM lactate)	0.38±0.028	n/a	1.7±0.1	[5]
Magnesium alloy foil	Porous platinum	Artificial tear (0.2mM lactate, 0.2mM glucose)	0.55±0.2	n/a	4.5±0.7	[6]
Au-CPE/GOx	Au-CPE/laccase	Artificial tear (20mM glucose)	0.53	2.3	3.18	[7]
LOx/TTF/GNRs	BOD/ABTS/GNRs	Artificial tear (3mM lactate)	0.58	97	18.6	This-work

TCP: Toray carbon paper; AuNPs: gold naparticles; TTF-TCNQ: tetrathialvalene tetracyanoquinodimethane; BP: buckypaper; poly-MG: polymerized methylene green; FcMe$_2$-LPEI: dimethylferrocene-modified linear polyethylenmine; An-pyr: anthracene-pyrene; MWCNT: multiwalled carbon nanotube; TBAB: tetrabutylammonium bromide; SC: single chamber

.

Table S2. List of detailed parameters of the CNT type used.

**Product name**		**MWNT 30-100nm (Multi-wall Nanotube)**		
**Batch**		**JC20210512**	**Pack**	200g
**Item**	**Unit**	**Quality Standard**		**Test Method**
**Appearance**	/	Black Powder		Visual Inspection
**Electron Micrograph**	/	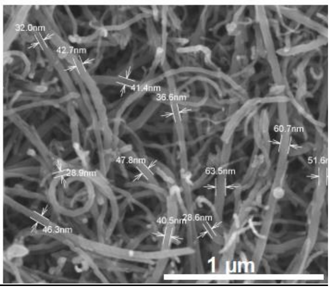		SEM（AIS2100C）
**Inside Diameter**	nm	5-15		
**Outside Diameter**	nm	30-100		
**Length**	μm	5-20		
**Interlamellar Spacing**	nm	0.34		
**Number of plies**	storey	30-50		
**BET surface area**	m^2/g	218.59		BET（SSA-3600）
**PH**	/	7.00-8.00		PH meter test（PB-10/C）
**Moisture Content**	%	0.5%		Moisture tester (MAC standard fast moisture tester)
**Ash Content**	%	0.2%		Carbon combustion method
**Ash (Cu, Mo, etc.)**	%	Mo	<0.1	ICP（Plasma1000 Inductively Coupled Plasma-Optical Emission Spectrometry）
	%	Mg	<0.1	
	Ppm	Cu	<1	
	Ppm	Fe	<1	
	Ppm	Ni	<1	
**Resistivity**	μΩ·m	965.4		After drying at 120 °C (the national standard is 105 °C), the pressure is 4MPa, the temperature is 23 °C, the humidity is 40-65%, and the current is 10mA. FZ-9601 resistivity meter.

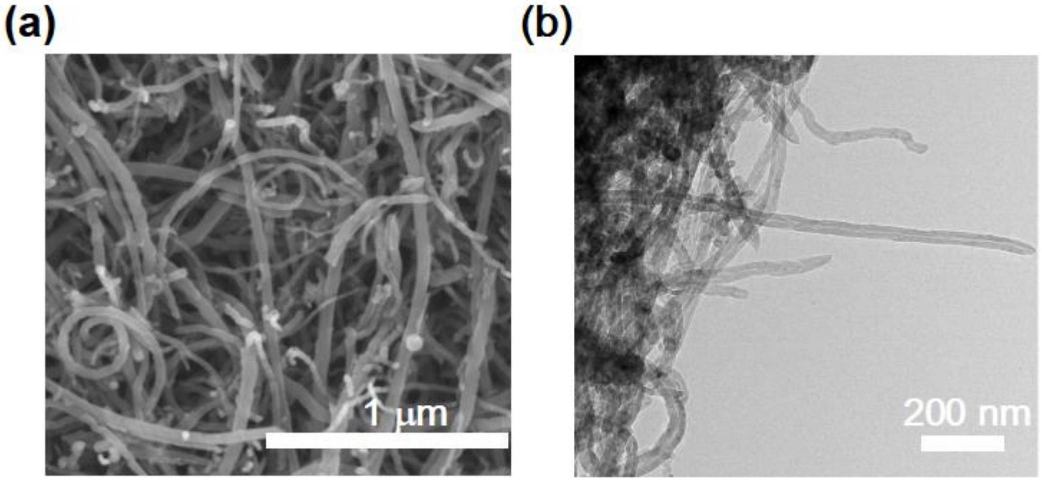

Figure S1. (a) SEM image of CNTs. (b) TEM image of CNTs.

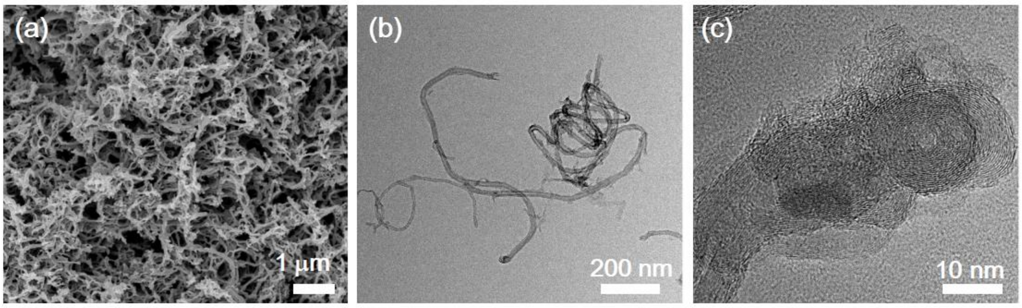

Figure S2. (a) SEM image of O-GNRs. (b) TEM image of O-GNRs. (c) HRTEM image of O-GNRs.

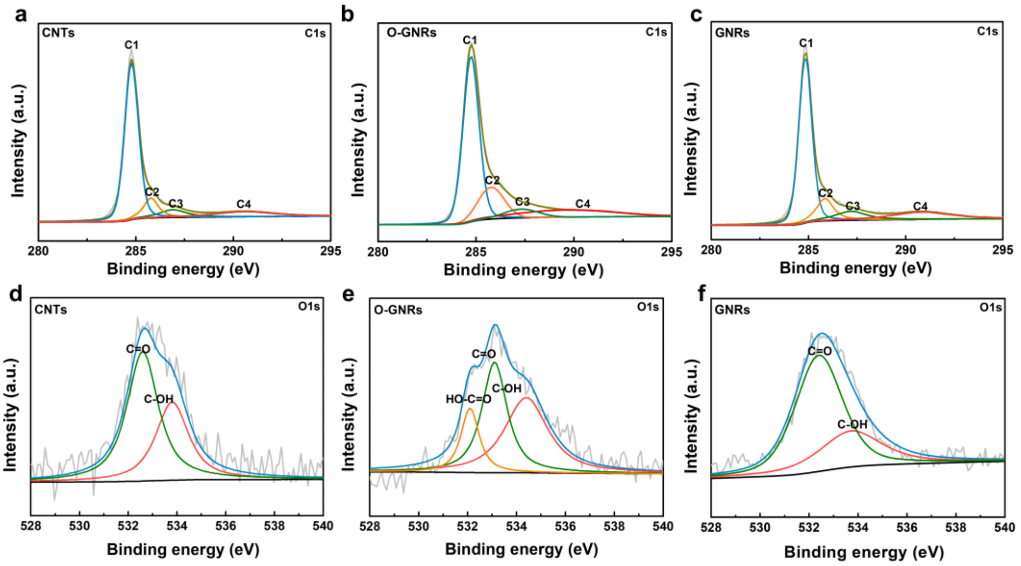

Figure S3. C 1s XPS spectra of CNTs (a), O-GNRs (b), and GNRs (c). O 1s XPS spectra of CNTs (d), O-GNRs (e), and GNRs (f).

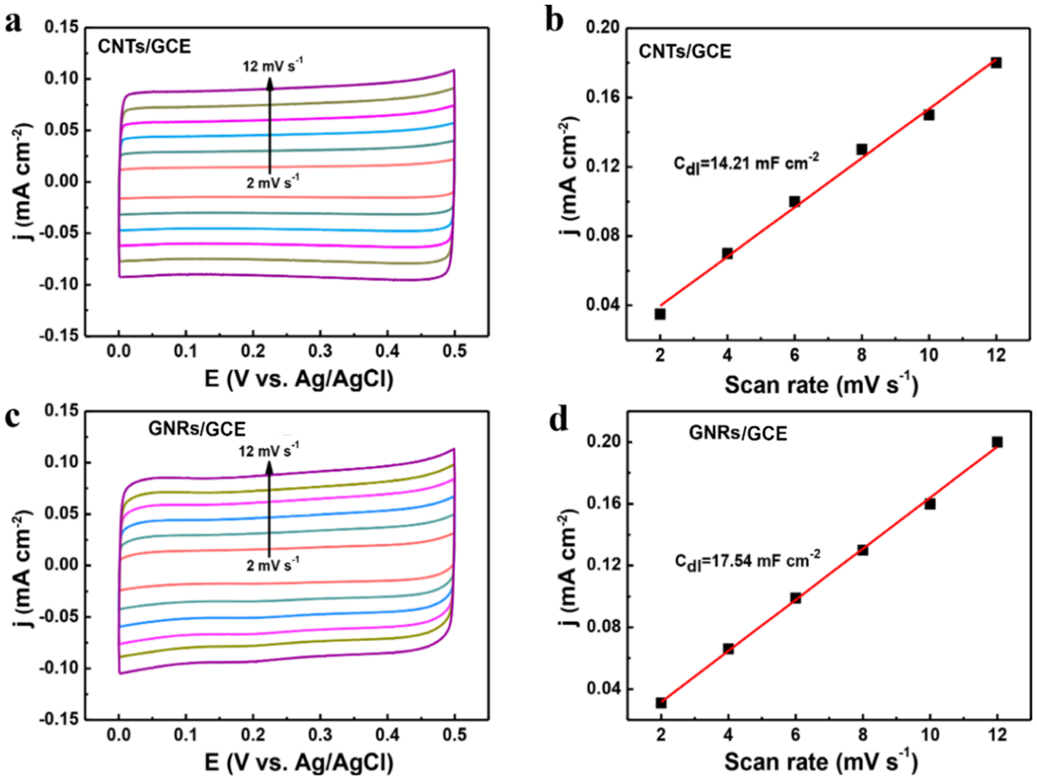

Figure S4. CVs curves of CNTs/GCE (a) and GNRs/GCE (c) in 0.1 M PBS (pH 7.2) at scan rate range of 2-12 mV/s. (b, d) The corresponding relationship of *j* vs. *v*.

The electrochemical active surface area (ECSA) is related to the double layer capacitance (C_{dl}) in the non-Faradaic potential region [8]. Typically, the larger the C_{dl}, the higher the ECSA. Specifically, C_{dl} was calculated based on a linear relationship between current density and different scanning rates (2, 4, 6, 8, 10, 12 mV/s). The C_{dl} was obtained by plotting (j_a-j_c) at 0.3 V vs. Ag/AgCl (where j_a represents anodic and j_c represents cathodic current densities, respectively) against the scan rates. The slope calculated by the above results was twice of C_{dl}.

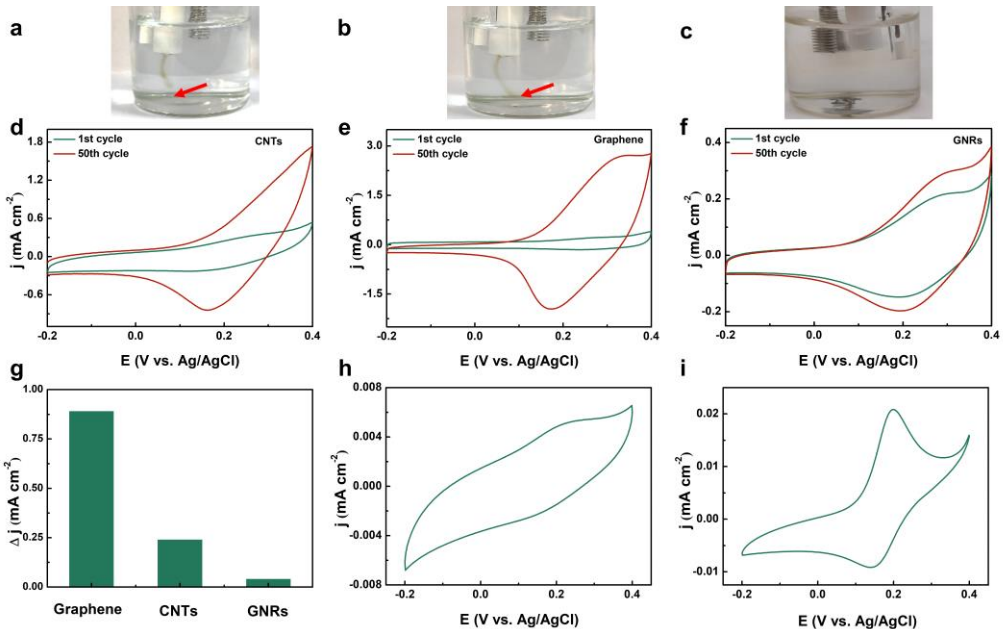

Figure S5. (a-c) Photographs of the electrolytic cells during CV measurement using (a) CNTs/GCE, (b) graphene/GCE, (c) GNRs/GCE as working electrode. CVs curves of (d) CNTs/GCE, (e) graphene/GCE, (f) GNRs/GCE for the 1st and 50th cycles. (g) The changed current values of the three materials between the 1st and 50th cycles at a constant potential of 0.2 V. (h) CVs curve of bare GCE in electrolyte after 50 cycles. (i) CVs curve of TTF loaded on GCE in 0.1 M PBS (pH 7.2) at a scan rate of 10 mV/s.

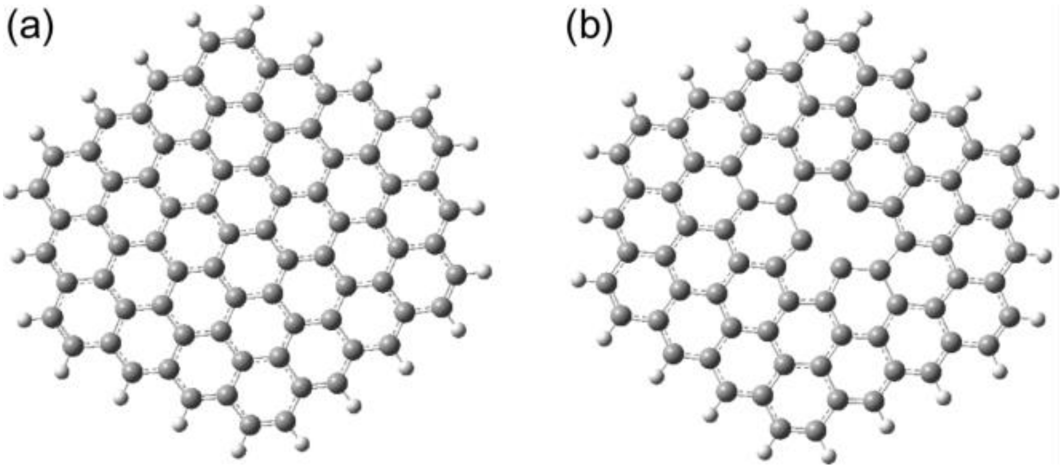

Figure S6. The structure of graphene and defective graphene.

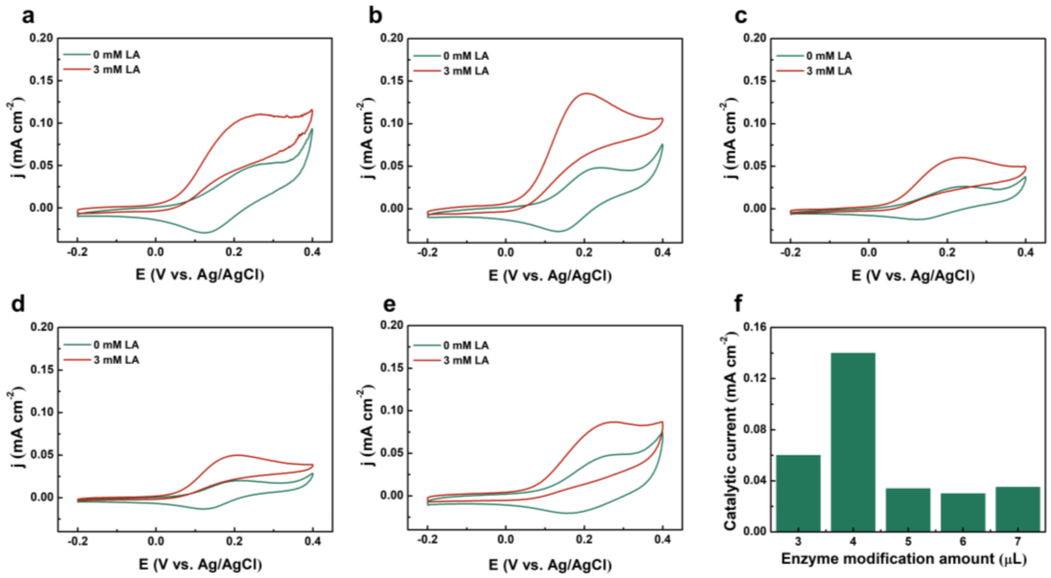

Figure S7. CVs curves of LOx/TTF/GNRs with different amounts of enzyme in 0.1 M PBS (pH 7.2): (a) 3 μL, (b) 4 μL, (c) 5 μL, (d) 6 μL, (e) 7 μL, at a scan rate of 10 mV/s. (f) The relationship between peak current and enzyme concentration.

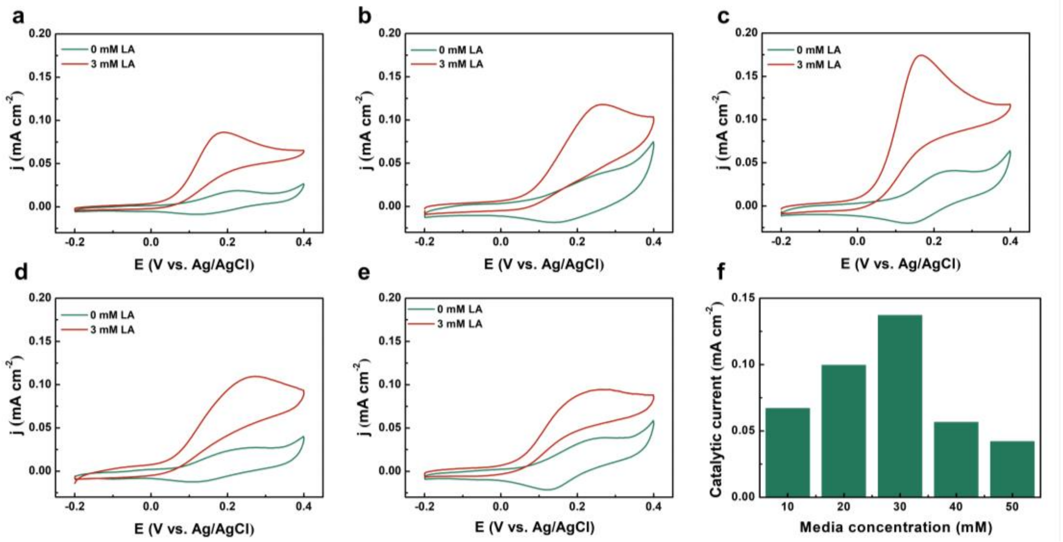

Figure S8. CVs curve of LOx/TTF/GNRs with different concentrations of TTF in 0.1 M PBS (pH 7.2) at a scan rate of 10 mV/s; (a) 10 mM, (b) 20 mM, (c) 30 mM, (d) 40 mM, (e) 50 mM. (f) The relationship between peak current and the mediator concentration.

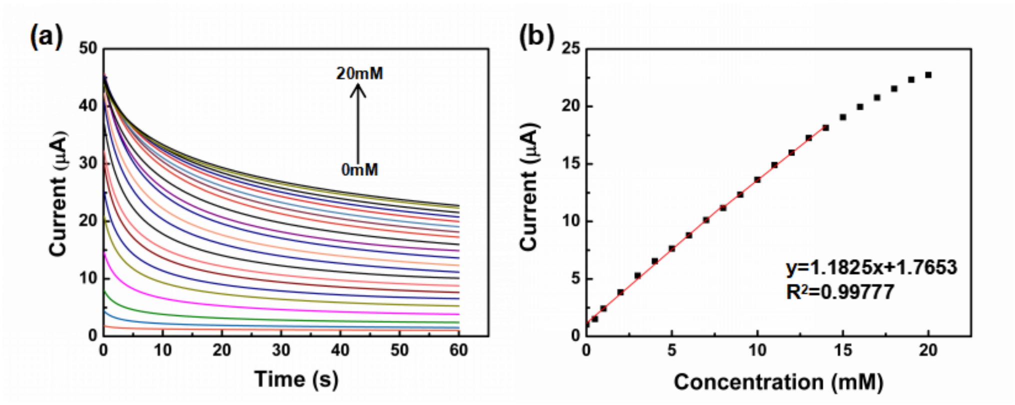

Figure S9. (a) CA curves of LOx/TTF/GNRs/GCE bioanode in the absence and presence of lactic acid with different concentrations with an applied potential of 0.2 V vs. Ag/AgCl. (b) Dependence of the catalytic currents on concentrations of lactic acid.

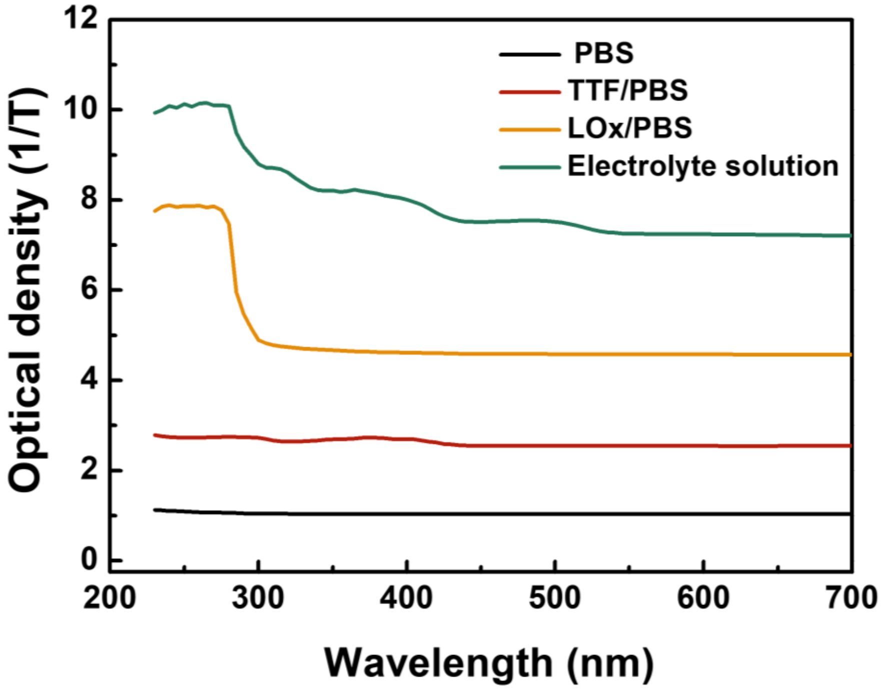

Figure S10. UV-vis diffuse absorption spectra of the electrolyte solution after 50 cycles, and 0.1 M PBS (pH 7.2) in the absence and presence of TTF or LOx.

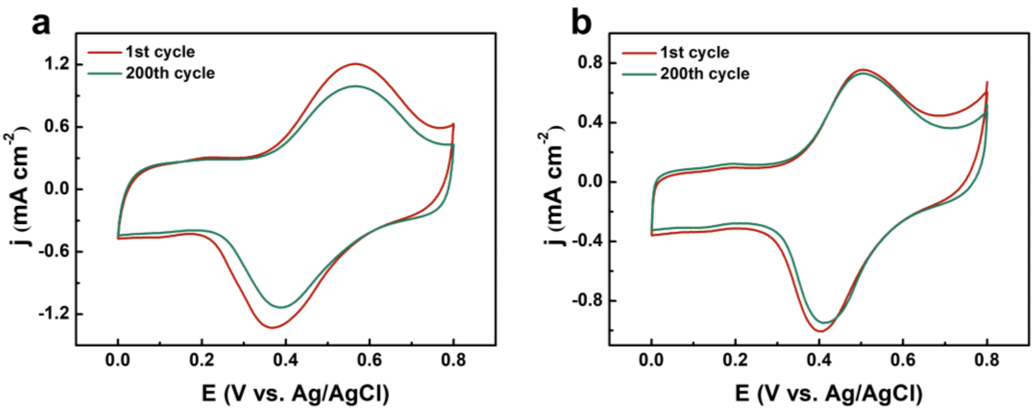

Figure S11. CVs curves of BOD/ABTS/GNRs/GCE biocathode in the absence (a) and presence (b) of Nafion protective layer at a scan rate of 10 mV/s.

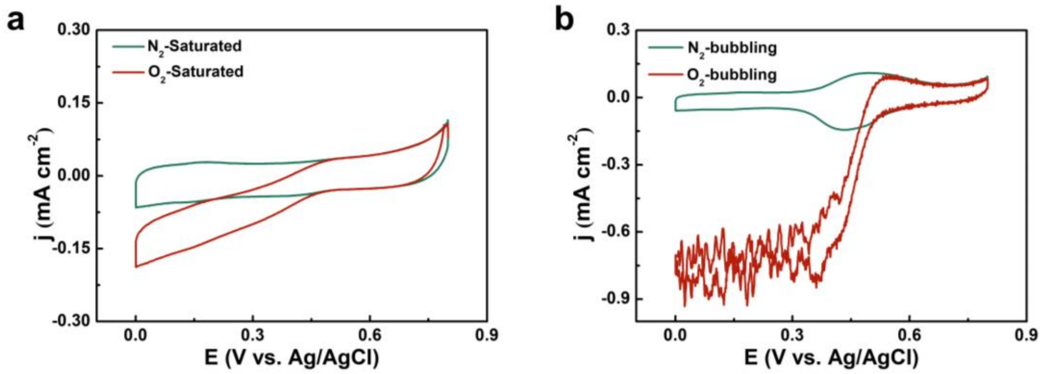

Figure S12. (a) CVs of BOD/GNRs/GCE biocathode in 0.1 M PBS buffer (pH 7.0) under N_2-saturated and O_2-saturated atmosphere at a scan rate of 10 mV/s. (b) CVs of BOD/GNRs/ABTS/GCE biocathode in 0.1 M PBS buffer (pH 7.0) under N_2-bubbling and O_2-bubbling atmosphere at a scan rate of 10 mV/s.

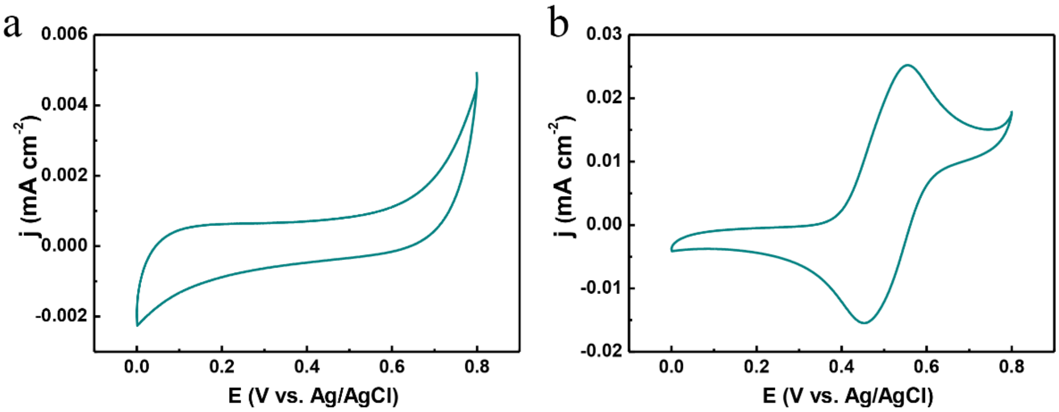

Figure S13. CVs curves of GCE in (a) the electrolyte after stability measurement of the cathode and (b) 0.1 M PBS (pH 7.2) containing 5 mM ABTS at a scan rate of 10 mV s^{-1}.

References

1. Azharuddin, M.; Bera, S.K.; Datta, H.; Dasgupta, A.K. Thermal fluctuation based study of aqueous deficient dry eyes by non-invasive thermal imaging. *Exp. Eye Res.* **2014**, *120*, 97–102.
2. Frei, M.; Martin, J. Power supply for electronic contact lenses: Abiotic glucose fuel cells vs. Mg/air batteries. *J. Power Sources* **2018**, *401*, 403–414.
3. Falk, M.; Andoralov, V.; Blum, Z.; Sotres, J.; Suyatin, D.B.; Ruzgas, T.; Arnebrant, T.; Shleev, S. Biofuel cell as a power source for electronic contact lenses. *Biosens. Bioelectron.* **2012**, *37*, 38–45.
4. Jayapiriya, U.S.; Goel, S. Flexible and optimized carbon paste electrodes for direct electron transfer-based glucose biofuel cell fed by various physiological fuids. *Appl. Nanosci.* **2020**, *10*, 4315–4324.
5. Reid, R.C.; Jones, S.R.; Hickey, D.P.; Minteer, S.D.; Gale, B.K. Modeling Carbon Nanotube Connectivity and Surface Activity in a Contact Lens Biofuel Cell. *Electrochim. Acta* **2016**, *203*, 30–40.
6. Falk, M.; Andoralov, V.; Silow, M.; Toscano, M.D.; Shleev, S. Miniature biofuel cell as a potential power source for glucose-sensing contact lenses. *Anal. Chem.* **2013**, *85*, 6342–6348.
7. Xiao, X.; Siepenkoetter, T.; Conghaile, P.O.; Leech, D.; Magner, E. Nanoporous Gold-Based Biofuel Cells on Contact Lenses. *ACS Appl. Mater. Inter.* **2018**, *10*, 7107–7116.
8. Wang, Z.; Ang, J.; Liu, J.; Ma, X.Y.D.; Kong. J.; Zhang, Y.; Yan, T.; Lu, X. FeNi alloys encapsulated in N-doped CNTs-tangled porous carbon fibers as highly efficient and durable bifunctional oxygen electrocatalyst for rechargeable zinc-air battery. *Appl. Catal. B Environ.* **2020**, *263*, 118344.
